# A Method for the Simultaneous Determination of Chlorogenic Acid and Sesquiterpene Lactone Content in Industrial Chicory Root Foodstuffs

**DOI:** 10.1155/2014/583180

**Published:** 2014-12-04

**Authors:** Honorine Willeman, Philippe Hance, Anne Fertin, Najia Voedts, Nathalie Duhal, Jean-François Goossens, Jean-Louis Hilbert

**Affiliations:** ^1^UMR 1281, Stress Abiotiques et Différenciation des Végétaux Cultivés, GIS GENOCHIC, INRA, IFR147, Université Lille 1 Sciences et Technologies, Cité Scientifique, 59655 Villeneuve d'Ascq, France; ^2^IUT A, Département Génie Biologique, Université Lille 1 Sciences et Technologies, 59655 Villeneuve d'Ascq, France; ^3^Faculté des Sciences Pharmaceutiques et Biologiques, Centre Universitaire de Mesures et d'Analyses (CUMA), Université Lille 2, BP 83, 3 rue du Pr. Laguesse, 59006 Lille Cedex, France

## Abstract

A method for the simultaneous determination of free chlorogenic acids (CGA) and sesquiterpene lactones (STL) in chicory root and its dried (flour) and roasted (grain) forms is described. The method uses one extraction and one analysis for all chicory root products. Various solvents with low to high polarity, such as methanol, chloroform, or *n*-hexane, were tested alone, in combination in different proportions or with acidified or neutral aqueous solvent. The water/chloroform/methanol (30/30/40, v/v/v) mixture generated the best extraction yield, 21% higher than alcohol mixtures. The profiling of CGA and STL content was performed through a conventional HPLC-DAD method using a PFP core shell column in a fast single run. Good retention time and area repeatability (RDD mean % 0.46 and 5.6, resp.) and linearity (*R* ^2^ ≥ 0.96) were obtained. The STL and chlorogenic acids levels determined were 254.7 and 100.2 *μ*g/g of dry matter in the root, 792.5 and 1,547 *μ*g/g in flour, and 160.4 and 822.5 *μ*g/g in the roasted grains, respectively. With an average recovery of 106% and precision of 90%, this method is rapid, reproducible, and straightforward way to quantify the chlorogenic acids and STL in chicory raw material and end products.

## 1. Introduction

Sesquiterpene lactones (STL) and chlorogenic acids (CGA) are soluble secondary main metabolites that accumulate in* Asteraceae*, especially in chicory [1]. The diversity of the chemical structure of these compounds allows these molecules to be involved in various physiological processes used by plants, such as the attraction of pollinators [2], the repulsion of herbivores [3], and protection against pathogens [4].

Many cultivated types of chicory (*Cichorium intybus* L.) exist, such as those used in salads, forage, and, in particular, industrial chicory utilized for the production of roots as raw material. Industrial chicory (*Cichorium intybus* var.* sativum*) has economic importance in many agricultural regions in the world as a source of inulin or food [5]. In northern France, chicory root is grown especially for use as dried or roasted products and beverage. Once harvested, the root is sliced and dried to produce an intermediate raw material called green slices. The slices are either ground to obtain flour or roasted and then crushed into grains. The grains can be marketed directly or extracted with hot water to obtain a concentrated liquid or, after spray-drying, soluble powders. Chicory flour can be used as bread-improving ingredient, and when roasted, chicory is used to enhance the aroma, color, or flavor of food. Thus, chicory root is consumed in different forms of derivative products in the human diet.

In addition to their known roles in plants, secondary metabolites present in chicory root may add value to food products via their biological properties. STL and CGA are known for their anti-inflammatory [6], analgesic [7], and neuroprotective actions [8]. 3,5-Di-*O*-caffeoylquinic acid, a chlorogenic acid, is responsible for nearly 70% of the antioxidant activity of chicory [9]. In addition to their potential beneficial effects on human health, these secondary metabolites play a role in the organoleptic properties of chicory. Indeed, chicory root is known for its bitter taste, which is essentially due to STL, mainly lactucin (Lc) and lactucopicrin (Lp) [10, 11].

Taken together, these nutritional and organoleptic properties are important factors for the appreciation and consumption of chicory products. The contents of STL and CGA represent a quality parameter of the raw material. Characterizing these compounds is part of a broader framework of varietal improvement and process optimization programs designed to improve the end product quality. However, these approaches are constrained by the requirement for the often tedious extraction of several thousand samples, routine analysis, and multiple assays to ensure the detection of these molecular families in various matrices.

The composition and distribution of STL and CGA have been extensively studied in leaf products of chicory crops such as salads, chicon, and forage [12–14]. However, despite studies of these compounds in fresh or roasted roots [11, 15, 16], the contents of both free STL and CGA in industrial chicory root and its derivative foods have not been determined.

Extraction modalities differ from one group of molecules to another and depend on the plant material from which the molecules are extracted. From the leaf of* Cichorium intybus* L. var.* sylvestre*, Mulinacci et al. [17] extracted CGA using water and fractionation by many solvents (ethanol,* n*-hexane, chloroform, and ethyl acetate). Price et al. [10] used methanol and chloroform to extract STL from the leaf of* Cichorium intybus* L. var.* foliosum* and var.* sylvestre*. Van Beek et al. [11] used ethyl acetate, methanol, chloroform, ethanol, and acetone sequentially to extract STL from the root of* Cichorium intybus*. More recently, the extraction of STL and CGA from “Catalogna and Head Radicchio” chicory leaves using a unique mixture of solvents was proposed [18] with separate analyses for the STL and CGA by high-performance liquid chromatography coupled to a diode array detector (HPLC-DAD).

Until now, no method for determining the major secondary metabolites involved in the qualities of the chicory root and processed products from this plant has been described. The purpose of this study was the development and validation of a method for the simultaneous determination of free STL and CGA from the root of* Cichorium intybus* var.* sativum* and its food products, including extraction and analysis. In our work, we tested different solvents and combinations of solvents to extract these major secondary metabolites from several matrices. We also developed a quantitative analytic method on conventional HPLC-DAD and a polar reverse-phase column Kinetex PFP for the rapid and self-limiting separation of target compounds. Compounds were authenticated by ultraperformance liquid chromatography coupled with high resolution mass spectrometer (UPLC-HRMS).

## 2. Methods

### 2.1. Biological Materials

Three major transformation products of industrial chicory were analyzed. The root was obtained from an agronomic trial, located in Coutiches, France, conducted in 2011 by the company Florimond-Desprez Veuve et Fils SAS (Capelle-en-Pévèle, France). Flour and roasted grains were obtained from the company Leroux SAS (Orchies, France).

The samples were immediately frozen in liquid nitrogen and stored at −80°C. For the experiments, the samples were freeze-dried (48 h) and ground (ball mill, Retsch, Eragny sur Oise, France) using 50 mL wells and 25 mm balls to obtain a fine powder. These samples were stored at −80°C.

### 2.2. Analytical Reagents and Chemicals

**Figure 1:**
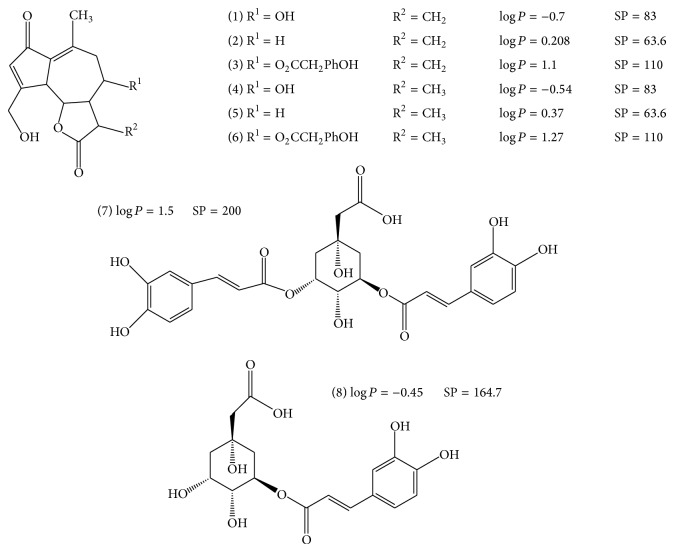
Structure, log⁡⁡*P* values, and surface polarity (SP) of the six sesquiterpene lactones: lactucin (1), 8-deoxylactucin (2), lactucopicrin (3), 11(S),13-dihydrolactucin (4), 11(S),13-dihydro-8-deoxylactucin (5), 11(S), 13-dihydrolactucopicrin (6), and two chlorogenic acids: 3,5-di-*O*-caffeoylquinic acid (7) and 5-mono-*O*-caffeoylquinic acid (8) identified in chicory roots products.

The solvents (methanol (MeOH), ethanol (EtOH), chloroform (CHCl_3_), dichloromethane (CH_2_Cl_2_), acetonitrile (ACN), acetone (Ac), ethyl acetate (EtOAc), and* n*-hexane (*n*-hex)), acetic acid (AAC), formic acid (FA), and* ortho*-phosphoric acid used for the extraction and chemical analysis were all HPLC grade and were obtained from the Dislab company (Lens, France). All of these solvents and acids were stored at 4°C. The standards used for the HPLC analysis were 5-mono-*O*-caffeoylquinic acid (5-CQA) and 3,5-di-*O*-caffeoylquinic acid (3,5-diCQA) and were provided by Biopurify (Chengdu, China). 11(S),13-Dihydrolactucin (DHLc), lactucin (Lc), 11(S),13-dihydrolactucopicrin (DHLp), and lactucopicrin (Lp) were provided by Extrasynthèse (Genay, France); 11(S),13-dihydro-8-deoxylactucin (DHdLc) and 8-deoxylactucin (dLc) were extracted, purified in the laboratory from industrial chicory root, and authenticated by ^1^H nuclear magnetic resonance (NMR) and ^13^C NMR [19] (Figure 1).

### 2.3. Extraction of CGA and STL

Several extraction methods have been tested using different solvents and combinations to extract CGA and STL simultaneously, including methanol 100% (MeOH); ethanol 100% (EtOH); acetone 100% (Ac); ethyl acetate 100% (EtOAc);* n*-hexane 100% (*n*-hex); chloroform 100% (CHCl_3_); dichloromethane 100% (CH_2_Cl_2_); 25/75, 50/50, and 75/25 mixtures (v/v) of water/methanol (H_2_O/MeOH), water/chloroform (H_2_O/CHCl_3_), and water/ethanol (H_2_O/EtOH); 75/24/1, 75/23/2, 75/22/3, and 75/21/4 mixtures (v/v/v) of methanol/water/acetic acid (MeOH/H_2_O/AAC) and methanol/water/formic acid (MeOH/H_2_O/FA); and 40/40/20, 30/30/40, 25/25/50, and 20/20/60 mixtures (v/v/v) of water/chloroform/methanol (H_2_O/CHCl_3_/MeOH). These extractions were carried out by maceration; 100 mg powder was extracted with 1.5 mL of solvent (1/15 ratio, w/v). For the extraction modalities with water/chloroform and water/chloroform/methanol, the solvents were added one at a time. The tubes were agitated for 17 h (o/n) in the dark at room temperature. The tubes were then centrifuged (12,000 r.p.m. at 4°C for 8 min), and the supernatants were filtered in 96-well microplates (Pall GHP 0.45 *μ*m, VWR, Fontenay-sous-Bois, France) and then analyzed by HPLC-DAD. Each extraction method was repeated three times. The H_2_O/CHCl_3_ and H_2_O/CHCl_3_/MeOH systems were immiscible, but adding 500 *μ*L of MeOH allowed for the phases mixing.

### 2.4. HPLC-DAD Quantification

A Prominence HPLC system (Shimadzu, Marne la Vallée, France) consisting of a quaternary pump (LC-20AD) and a UV-visible diode array detector (SPD-20A) was used to detect CGA and STL at 320 and 254 nm, respectively. A Kinetex PFP column (100 × 4.6 mm, 2.6 *μ*m) (Phenomenex, Le Pecq, France) was used for compound separation. The separation method lasted 16.5 min. The elution was performed in gradient mode using three different solvents—water (solvent A), MeOH (solvent B), and ACN (solvent C)—all acidified with 0.1%* ortho*-phosphoric acid. Solvent B gradient rose from an initial 7.5–17.5% at 1 min, 32.5% at 5 min, and 80% at 8 min, returning to 7.5% at 9 min until the end of the run; solvent C was kept at 12% throughout. The oven temperature was 45°C, and the flow rate was 1.1 mL/min. The amount of sample injected was 5 *μ*L.

The quantification was performed by external calibration using standards (5-CQA, 3,5-diCQA, DHLc, Lc, DHdLc, dLc DHLp, Lp.), each repeated five times. The respective calibration curves were constructed by linear regression plotting signal area versus compound concentration. The analytic precision was measured within and between days. Each extract was analyzed three times consecutively and three times on three different days.

### 2.5. UPLC-High Resolution MS Analysis/Authentication

Chromatographic separation was performed on an Accela UPLC system (Thermo Scientific) equipped with a Kinetex PFP column (100 × 4.6 mm, particle size 2.6 *μ*m; Phenomenex). The elution was performed with the same HPLC-DAD gradient mode as described previously using water (solvent A), MeOH (solvent B), and ACN (solvent C), all acidified with 0.1% formic acid. The LC flow rate was 1 mL/min; the injection volume was 5 *μ*L. The column oven temperature was 45°C and the sample tray temperature 4°C.

Eluted compounds were detected in negative mode in full mass scan (*m/z* 120 to 900) using a Thermo Scientific Orbitrap Mass Spectrometer Q Exactive equipped with a heated electrospray ionization source (HESI-II). Instrument parameters were as follows: sheath gas 60, auxiliary gas 20 (both arbitrary units), spray voltage 4 kV, capillary temperature 275°C, capillary voltage −60 V, tube lens voltage −135 V, skimmer voltage −20 V, and source temperature 300°C.

Mass spectra were recorded at a resolution of 50,000 with an automatic gain control (AGC) target of 500,000 and a maximum injection time of 500 ms.

### 2.6. Validation Method

#### 2.6.1. Linearity, Limit of Detection (LOD), and Limit of Quantification (LOQ)

Linearity was studied for each molecule. Using a series of seven dilutions, the standard concentrations ranged from 3.3 to 70,000 ng/mL, and each was repeated five times.

The LOD and LOQ were determined for each molecule. All dilutions were analyzed by HPLC-DAD to determine the average concentration (*μ*) corresponding to the loss of signal detection and quantification (blank sample) for each of the three products. The LOD was defined by *μ* + 3*σ* and LOQ by *μ* + 10*σ*, where *σ* represents the standard deviation of the blank.

#### 2.6.2. Precision and Specificity

Repeatability was evaluated for each product by six successive repetitions of the extraction method. Reproducibility was calculated for each product by three successive repetitions performed on different days. The coefficient of variation (% respective standard deviations (RSD)) served as a measure of precision.

The specificity of the extraction method was evaluated by varying the proportions of solvents H_2_O/CHCl_3_/MeOH plus and minus 4% compared to the optimal method.

#### 2.6.3. Extractability and Recovery Rate Matrix Effect

The root, flour, and roasted grains were extracted using the optimum conditions according to the extraction method (see Section 2.3). Once all the supernatant was recovered, the pellet was reextracted under the conditions described above. Three repetitions were performed on each product. The extraction efficiency was the rate of extractable compounds (without reextraction) under the optimal modality and was measured as the ratio between the contents obtained during the first extraction (*E*1) and all contents (first extraction (*E*1) + reextraction 17 h (*E*2)). The extraction rate (ER) is expressed as

(1) $$\begin{array}{c} \text{ER}=\left(\frac{E1}{\left(E1+E2\right)}\right)\times 100. \end{array}$$

The recovery rate was calculated by adding 0.075, 0.15, or 0.3 mM of 5-CQA; 0.0075, 0.03, or 0.075 mM of 3,5-diCQA; or 0.007, 0.014, or 0.045 mM of DHLc to the flour. Three replicates are performed by employing the optimum extraction modality. The recovery rate (RR) is the ratio between the measured content (MC) and the theoretical content (TC):

(2) $$\begin{array}{c} \text{RR}=\left(\frac{\text{MC}}{\text{TC}}\right)\times 100. \end{array}$$

The effect of the extraction mixture on possible conversions of the chemical structures of the target compounds was evaluated by observing the variation in their content by HPLC-DAD analysis. Three representative target compounds (5-CQA, 3,5-diCQA, and DHLc) were directly added individually in three different concentrations to the solvent mixture, in a chicory or “no-chicory matrix” (flour wheat). The extraction procedure was completed as described. The overlapping of chromatographic signals corresponding to the addition of the molecules was evaluated. No matrix effect was obtained when no interfering peak was present and when the linearity of the response was observed.

### 2.7. Statistics and Calculation

All extractions and analyses were performed in triplicate, except for linearity, which was repeated five times. Data are expressed as the means of individual repeats with the RSD of each extraction condition. Statistical analysis was performed using R 2.15.1 for Windows [20] and used to examine between-extract variation. When the Bartlett test was significant for homogeneity of variance, the extraction effect on metabolite levels was estimated by a one-way ANOVA, and mean separations were evaluated by pairwise comparison using Student's *t*-test. Log *P* values (predicted lipophilicity) were calculated from online cheminformatics services provided by the Molinspiration Property Calculation Service (2013) [21].

## 3. Results and Discussion

### 3.1. Method Development

Several methods for extracting STL or CGA from the leaves of* Cichorium intybus* L. var. Catalogna and var. Rosso di Chioggia [18], var.* sylvestre* [17], and* Cichorium endivia* var.* crispa* [22] have been described. However, no method for the determination of STL and CGA exists for industrial chicory root and its derivatives. In our work, these compounds were determined in different products of chicory from a single extraction and analysis. The relative contents obtained from the extraction were evaluated by HPLC-DAD.

The first screen was performed using pure solvents that cover a wide range of polarity, including MeOH, EtOH, Ac, EtOAc,* n*-hex, CHCl_3_, or CH_2_Cl_2_. Only the contents obtained from a selection of solvents (maximum extraction without excluding any metabolites) are shown in Table 1. Thus, the extraction efficiency of MeOH compared to EtOH or CHCl_3_ was, on average, 4.5 times higher for all products. In general, in terms of the quantity of STL and CGA extracted, methanol is more effective (*P* value < 0.05) than the less polar solvents such as chloroform [23] and ethanol [24]. The other tested solvents, including acetone, ethyl acetate,* n*-hexane, and dichloromethane, delivered extraction yields up to 7 times lower than MeOH. These data are confirmed by the work of Ferioli and D'Antuono [18], which demonstrated that the extraction of chicory salad with MeOH yields at least twice the STL of extraction with EtOH, acetone, or ethyl acetate. Although molecules are mainly extracted with chloroform, ethanol, and methanol, the content increases with solvent polarity. The solubility of STL and CGA, such as Lc or 5-CQA, in the alcoholic solvents may be due to the high surface polarity notably induced by hydroxyl, carbonyl, and *γ*-lactone moieties. However, the presence of methyl, methylene, and phenyl groups provides a lipophilic character to the compounds that can explain their extractability in less polar solvents, such as chloroform. As observed with DHLp and Lp, their greater solubility in chloroform and in nonaqueous solvents, such as hexane or dichloromethane (data not shown), might be related to their higher lipophilicity shown by the calculated log⁡⁡*P* values of 1.27 and 1.1, respectively (Figure 1).

Extractability was increased by expanding the polarity range with the addition of water (25, 50, or 75%) to three selected solvents. The aqueous alcohol system H_2_O/MeOH (25/75, v/v) was more effective in extracting compounds with a low affinity for alcohol. Indeed, the addition of water increased the extraction efficiency of MeOH by 1.4, 1.5, and 2.2 times for the root, roasted grains, and flour, respectively (Table 1). The water simultaneously reduced the dehydrating effect of MeOH and promoted its diffusion in the matrix, allowing a better penetration of the solvent mixture and an increased extraction of STL. These actions help to solubilize all target compounds more extensively. However, the effect was limited and resulted in a loss of performance when the percentages of water reached 50 and 75%. Indeed, according to Elliott et al. [25], the swelling of the membranes favors the penetration of alcohol and is optimal when the proportion of water in the aqueous alcohol mixture is 25%. These results are confirmed by Song et al. [24], who found that a porous membrane is favored by a high concentration of alcohol. Under the same conditions, the water was tested in combination with CHCl_3_ or EtOH. As in the case of MeOH, the best extraction rates were achieved with 25% water. Whereas a mixture of water and ethanol did not improve the extraction efficiency of the H_2_O/MeOH system (25/75, v/v), adding water to CHCl_3_ increased the extraction rate of the roasted product and root by 2- and 3-fold, respectively. Chloroform, which is less polar than water, carries more amphiphilic-like and fewer polar compounds. According to Burianek and Yousef [26], the interface between these two solvents, which results from their immiscibility, forms a suitable environment to concentrate these molecules. Moreover, during maceration, agitation optimizes not only the transfer of matrix molecules to water but also water to this interface. Therefore, the molecules' affinity for less polar phases promotes their extraction in chloroform. To increase the extraction polarity, optimization was conducted using an H_2_O/MeOH combination (25/75, v/v), which we denote as H_2_O/MeOH.

Organic acids are known to penetrate and destabilize agent membranes [27]. As such, acetic acid (AAC) and formic acid (FA) were tested in addition to the methanol mixture to maximize extraction. The MeOH/H_2_O/AAC system (75/24/1, v/v/v) conferred the same extraction yields for the root and flour but up to 1.5 times lower than the H_2_O/MeOH system for the roasted grains (Table 1). The final proportion of acid in the mixture (2, 3, or 4%) or the use of formic acid instead of acetic acid did not cause a significant difference in the extraction yields of flour (*P* value = 0.06) and roasted grains (*P* value = 0.14). Furthermore, the superposition and similarity of the absorption spectra of target signals indicated no difference between an acidified extraction and a nonacidified extraction (data not shown).

The extraction method was optimized by combining water with chloroform and methanol in different proportions. The presence of chloroform in the mixture increased the extraction yield of the flour by 35% compared to H_2_O/MeOH (Table 1). Chloroform, a solvent of intermediate polarity, leads to the generation of twice as many amphiphilic-like molecules as H_2_O/MeOH, similarly to dLc and 3,5-diCQA. The presence of methanol in the water and the interface between chloroform and the aqueous phase would extend the polarity of the mixture, facilitating the extraction of polar and less polar compounds. These compounds were then recovered together by removing the interface through the addition of methanol, allowing their simultaneous analysis.

### 3.2. Authentication

Main secondary metabolites of the chicory are authenticated in chicory products by UPLC-HRMS.  Table 2 shows authentication characteristics of STL and CGA in flour. Theoretical exact mass (M) was calculated from the formula at which one proton is subtracted (M-H+) for negative mode analysis. Each molecular ion (M-H+) was extracted from the MS-TIC (Total Ionic Courant).

Exact mass (Mc), elementary formula, ring double bond (RDB), and precision (ppm) are calculated for each analyte. All elementary formula propositions with precision more than 7 ppm are rejected. For flour, the lowest ppm is 4.156 ± 0.260 for 3,5-diCQA and the highest is 6.658 ± 0.160 for DHLp, with an average of 5.866. When exact mass with exactly elementary formula and low ppm have the same RDB theoretical, we can authenticate the molecule. Each exact mass (Mc) is associated with a retention time (RT) and elution order is the same as for the HPLC-DAD analysis.

All metabolites are authenticated in flour (Table 2) but also in root and roasting grains (data not shown).

### 3.3. Validation

#### 3.3.1. LOD, LOQ, and Linearity

In this study, the following STL and CGA commonly found in chicory were studied: DHLc, Lc, DHdLc, dLc, DHLp, Lp, 5-CQA, and 3,5-diCQA. The quantification of target compounds was undertaken by an HPLC-DAD analysis of standards. For the efficient separation of all metabolites of interest while providing a meaningful flow analysis, we used chromatographic columns based on core-shell particles of 2.6 *μ*m [28]. A Kinetex PFP column (Phenomenex) was chosen because of its ability to separate positional isomers [29]. The samples analyzed showed separation with a very good resolution of the target compounds in 16.5 min. This method allows for the analysis of approximately 90 samples per day operating in a conventional chromatographic system.

Table 3 shows the RT, the LOD and LOQ, the linearity range, the coefficient of determination (*R* ^2^), and the equation of the slope of the standard range for each metabolite tested. The STL absorbed in UV range between 190 and 260 nm (max. 254 nm) and the CGA between 210 and 320 nm (max. 320 nm). The RTs of each molecule for all wavelengths were different, indicating the lack of coelution of molecules, as evidenced by the resolution factor Rs value above 8.5 (i.e., DHLp and Lp), promoting better quantization. This separation is even more effective using the Kinetex column, whose chemistry (based on PFP groups, aromatic interaction *π*-*π*, and H-F) allows for the resolution of positional isomers, which include the targeted CGA and STL. The analytic precision (repeatability and reproducibility) was measured by the intra- and interday (three different days) repetition method and is expressed in terms of the variation (RSD %) of RT and areas obtained (Table 4). A small variation of RT (here, a maximum of 0.9%) is very important to avoid the misidentification of peaks in samples. The area variation was, in general, small but higher for the reproducibility tests, with means of 6.8% for CGA and 6.4% for STL, as summarized in Table 4.

The LODs defined for all compounds were between 2 and 9.5 ng/mL (Table 3). The LOQs ranged from 3.3 to 89.5 ng/mL. Linearity ranges varied from 1 to 6,140 times the LOQ (Table 3). Thus, the concentration range, for all compounds, extended from 3.3 to 70,000 ng/mL. Linearity was perfectly preserved for each range, as illustrated by *R* ^2^ above 0.96. For each molecule, the H_2_O/CHCl_3_/MeOH system (30/30/40, v/v/v) enables the extraction of a content greater than the limit of quantification without surpassing the limit of linearity. Under these extraction conditions, no additional preparation, such as dilution or enrichment, is necessary for these samples.

#### 3.3.2. Precision and Specificity

The precision of the H_2_O/CHCl_3_/MeOH (30/30/40, v/v/v) extraction method was evaluated by six successive intraday repetitions and three successive interday repetitions. This variable is expressed in terms of variation (RSD %) by the repeatability and reproducibility of the obtained contents of each target metabolite (Table 4). A maximum of 10 and 12% variation was observed for repeatability and reproducibility, respectively. These results indicate good accuracy of the extraction for all target metabolites.

A variation of ±4% in the ratio of the H_2_O/CHCl_3_/MeOH solvents relative to the optimal modality (30/30/40, v/v/v) introduces a variability of ±20% from the average (data not shown). These results demonstrate the selectivity of the method and the importance of respecting the parameters set forth above.

#### 3.3.3. Extractability, Recovery Rate, and Matrix Effect

The extraction efficiency of the ternary mixture H_2_O/CHCl_3_/MeOH (30/30/40, v/v/v) was evaluated for each metabolite in the three products (Table 4). Residual contents measured from the reextraction of the pellet were used to define the extractability rate of the first method. The minimum extractability was observed for 3,5-diCQA in root (76%), and the maximum extractability was observed for the 5-CQA in the root (94%). For all metabolites, only one extraction of 17 h yielded 84% total efficiency for the roots and flour and 88% for the roasted grains. Extractability is favorable for STL present in roasted matrix, as shown by a yield close to 89%. However, the measured levels indicate a low initial concentration of STL in the roasted grains.

5-CQA, 3,5-diCQA, and DHLc were added to the flour at different concentrations to determine the recovery rate of the H_2_O/CHCl_3_/MeOH (30/30/40, v/v/v) extraction method. The flour was used because of its richness in STL and CGA. The concentrations tested were 0.075, 0.15, and 0.3 mM 5-CQA; 0.0075, 0.03, and 0.075 mM 3,5-diCQA; and 0.007, 0.014, and 0.045 mM DHLc. Recovery rates were between 94 and 122% (Table 5), with an average rate of 106%, indicating the effectiveness of the method.

The addition of different concentrations of 5-CQA, 3,5-diCQA, and DHLc in a chicory (flour) and “no-chicory” (wheat flour) matrix was used to evaluate the effect of the H_2_O/CHCl_3_/MeOH (30/30/40, v/v/v) extraction mixture on the conversion of the chemical structure of the target compounds. The choice of “no-chicory” material focused on wheat flour because the targeted metabolites are absent. In the chicory matrix, the variation in the levels of metabolites, with or without addition, was between 2 and 9% and was included within the repeatability values. In the “no-chicory” matrix, no conversion was observed for the other molecules (contents), and the solvent alone did not change the structure of the compounds (data not shown).

### 3.4. STL and CGA in Products Derived from Chicory

**Figure 2:**
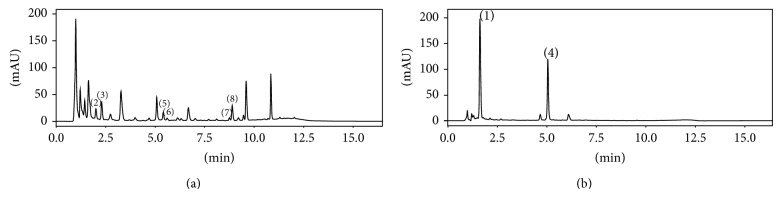
HPLC-DAD chromatograms of sesquiterpene lactones and chlorogenic acids in chicory root product (flour) obtained with H_2_O/CHCl_3_/MeOH 30/30/30 (v/v/v) extraction: (a) STL at 254 nm and (b) CGA at 320 nm. (1) 5-Mono-*O*-caffeoylquinic acid, (2) 11(S),13-dihydrolactucin, (3) lactucin, (4) 3,5-di-*O*-caffeoylquinic acid, (5) 11(S),13-dihydro-8-deoxylactucin, (6) 8-deoxylactucin, (7) 11(S), 13-dihydrolactucopicrin, and (8) lactucopicrin.

The flour and the roasted product were derived from the same batch of roots and were analyzed with the evaluated determination method (Figure 2). For the first time, the CGA and STL composition was demonstrated in chicory flour and the presence of STL in the roasted grains was identified. The sesquiterpene lactones and chlorogenic acids levels were 254.7 and 100.2 *μ*g/g of dry matter in the root, 792.5 and 1,547 *μ*g/g in flour, and 160.4 and 822.5 *μ*g/g in the roasted grains, respectively (Table 1). The ratio of secondary metabolites between the root and the flour was 5.7 (*P* value = 0.0014), between the root and the roasted grains 2.2 (*P* value = 0.0002), and between the flour and the roasted grains 2.6 (*P* value = 0.0021). The drying of roots increases the levels of metabolites, most likely due to the release of the bound forms, while roasting tends to degrade all molecules. Supplementary work is being conducted on the presence of the conjugated and free forms of the metabolites of interest. The study of the process effects on the content of secondary metabolites related to the organoleptic characteristics of the various products is also in progress.

## 4. Conclusion

Our work has shown that the ternary mixture H_2_O/CHCl_3_/MeOH (30/30/40, v/v/v) can be used to extract the content of chlorogenic acids and sesquiterpene lactones of chicory root and its main food derivatives, flour, and roasted grains, simultaneously, with a higher efficiency than aqueous alcohol systems and a strong representation of the molecules. Direct extraction of the material, without an intermediate fractionation step (liquid-liquid extraction, SPE), evaporation, or resolubilization, followed by a short and simple chromatographic analysis, is a flexible, fast, and effective method. The chromatographic method profiled eight molecules belonging to families of major soluble secondary metabolites of chicory plant and is conducted with one extraction and one analysis. This method of simultaneous determination allows for comparative studies (physiological, genetic, or influencing the process) among the many products derived from chicory.

This targeted approach is currently being evaluated with a more comprehensive approach in which different mixtures tested to achieve this optimal system, including water/chloroform/methanol solutions at 20/20/60 or 30/30/40 (v/v/v), are combined with methodologies developed for the analysis of plant metabolomics [30]. These studies suggest the possibility of extending the metabolomics approach we have developed for chicory.

## Figures and Tables

**Table 1:** Contents of CGA (5-CQA, 3,5-diCQA) and STL (DHLc, Lc, DHdLc, dLc, DHLp, Lp) in the root, flour, and roasted grains extracted by different solvents and solvent mixtures.

Solvents/solvent mixtures^a^	Metabolites^b^-*μ*g/g of dry matter ± SD									*P* value
	5-CQA	3,5-diCQA	DHLc	Lc	DHdLc	dLc	DHLp	Lp	TOTAL	
**Root**										
MeOH	52.5 ± 15.8^bc^	41.3 ± 12.9^ab^	26.7 ± 8.5^b^	52.8 ± 15.7^bc^	37.9 ± 3.7^ab^	3.6 ± 0.2^c^	3.8 ± 1^cd^	36.4 ± 2.7^c^	255 ± 56.1	2.30*E* − 02
EtOH	4.3 ± 0.7^d^	9.7 ± 2.2^c^	3.8 ± 0.5^c^	8.9 ± 1^d^	8.4 ± 1.3^d^	0.9 ± 0.05^d^	2.2 ± 0.1^c^	13.1 ± 1.1^d^	51.3 ± 6.8	—
CHCl_3_	3.6 ± 0.3^d^	1.9 ± 0.4^d^	4.8 ± 0.4^c^	8.6 ± 0.5^d^	4.7 ± 0.2^e^	1 ± 0.07^e^	1.4 ± 0.2^d^	9.8 ± 0.4^e^	35.8 ± 0.8	5.83*E* − 02
H_2_O/MeOH	66.2 ± 0.9^c^	37.4 ± 0.7^b^	48.7 ± 2.4^a^	88.6 ± 3.2^ab^	39.4 ± 0.3^b^	5.6 ± 0.06^a^	20.9 ± 0.6^a^	38.3 ± 0.07^c^	345.1 ± 6.5	7.14*E* − 07
MeOH/H_2_O/AAC	73.5 ± 1^b^	50.4 ± 0.8^a^	42.5 ± 1.5^b^	77.7 ± 2.8^c^	42.4 ± 1.2^a^	4.5 ± 0.4^b^	6.1 ± 0.4^b^	44.7 ± 1.5^b^	341.8 ± 7.8	1.29*E* − 06
H_2_O/CHCl_3_/MeOH	91.9 ± 5.7^a^	8.3 ± 0.7^c^	53.3 ± 3.3^a^	97.4 ± 5.5^a^	24.3 ± 0.3^c^	6.8 ± 1.3^ab^	18.9 ± 1.2^a^	54 ± 3.3^a^	354.9 ± 15.3	1.30*E* − 04

**Flour**										
MeOH	239.7 ± 29.3^c^	131.9 ± 19.2^c^	56.1 ± 5.3^d^	58.7 ± 10.9^d^	115.8 ± 17.4^c^	5 ± 0.7^c^	10.8 ± 0.9^b^	48 ± 8.5^cd^	666 ± 91.2	1.13*E* − 02
EtOH	35.8 ± 3.2^e^	46.8 ± 3.9^d^	15 ± 2.1^f^	46.3 ± 6.4^d^	59.4 ± 6.9^d^	3.6 ± 1^c^	7.8 ± 1.4^c^	58.5 ± 5.8^c^	273.2 ± 30.7	—
CHCl_3_	107.7 ± 11.6^d^	58 ± 2.5^d^	30.3 ± 1.5^e^	39.5 ± 1.5^d^	64.4 ± 2.9^d^	3.7 ± 0.1^c^	7.5 ± 0.08^c^	38.8 ± 0.5^d^	349.9 ± 18.1	2.63*E* − 02
H_2_O/MeOH	590.9 ± 21.3^b^	281.4 ± 16.3^b^	117.7 ± 3.5^b^	125.1 ± 4^b^	240.5 ± 8.6^b^	19.4 ± 1.4^b^	22.8 ± 2.2^a^	87.4 ± 7.2^ab^	1,485.2 ± 64.5	7.44*E* − 05
MeOH/H_2_O/AAC	557.6 ± 17.9^b^	300.2 ± 11.9^b^	104.3 ± 1.1^c^	103.8 ± 5.3^c^	235.8 ± 11.6^b^	29.3 ± 1.5^a^	19 ± 1.1^a^	83.6 ± 4^b^	1,433.6 ± 54.4	2.86*E* − 05
H_2_O/CHCl_3_/MeOH	810.7 ± 24.8^a^	410.6 ± 38.3^a^	147.8 ± 7.3^a^	150 ± 1^a^	318.8 ± 29.7^a^	38.3 ± 6.5^a^	27.2 ± 3.8^a^	110.4 ± 11.8^a^	2,013.8 ± 123.2	7.57*E* − 04

**Roasted grain**										
MeOH	345.4 ± 38.7^c^	79.4 ± 8.4^c^	3.1 ± 1.1^a^	32.3 ± 3.4^a^	43.9 ± 4.3^c^	5.2 ± 3.3^b^	4.4 ± 0.9^a^	22 ± 1.4^b^	535.7 ± 61.5	3.51*E* − 03
EtOH	56.5 ± 4.5^d^	20.2 ± 3.4^d^	0.3 ± 0.1^b^	14.4 ± 1.7^d^	16.1 ± 3.3^d^	nd	0.5 ± 0.7^cd^	12.5 ± 0.9^c^	120.5 ± 14.6	—
CHCl_3_	37.5 ± 14.7^d^	16.9 ± 1.3^d^	0.3 ± 0.3^b^	13.1 ± 1.8^d^	12.9 ± 0.8^d^	nd	2.3 ± 0.2^b^	11.6 ± 1.3^c^	94.6 ± 20.4	9.73*E* − 02
H_2_O/MeOH	564 ± 28.3^a^	143.4 ± 9.1^a^	nd	22.1 ± 3.6^bc^	64.2 ± 3.5^ab^	nd	nd	26.7 ± 4.2^ab^	820.4 ± 48.7	2.44*E* − 04
MeOH/H_2_O/AAC	375.4 ± 8.1^c^	92.6 ± 9.1^c^	nd	16.5 ± 1.6^cd^	61.7 ± 8.4^b^	nd	1 ± 0.07^d^	18.2 ± 0.8^a^	565.4 ± 28.1	1.87*E* − 05
H_2_O/CHCl_3_/MeOH	499.6 ± 22.8^b^	119.2 ± 10.4^b^	1.3 ± 2.3^ab^	22.9 ± 0.1^b^	92.8 ± 7.9^a^	16.4 ± 5.4^a^	1.3 ± 0.09^c^	25.7 ± 3.3^ab^	779.2 ± 51.9	6.96*E* − 06

Different letters in the same column within the same product indicate significant differences (*P* < 0.05).

*P* value: calculated by comparison to the results obtained with ethanol extraction.

^a^MeOH: methanol 100%; EtOH: ethanol 100%; CHCl_3_: chloroform 100%; H_2_O/MeOH: water/methanol 25/75 (v/v); MeOH/H_2_O/AAC: methanol/water/acetic acid 75/24/1 (v/v/v); H_2_O/CHCl_3_/MeOH: water/chloroform/methanol 30/30/40 (v/v/v).

^b^5-CQA: 5-mono-*O*-caffeoylquinic acid; 3,5-diCQA: 3,5-di-*O*-caffeoylquinic acid; DHLc: 11(S),13-dihydrolactucin; Lc: lactucin; DHdLc: 11(S),13-dihydro-8-deoxylactucin; dLc: 8-deoxylactucin; DHLp: 11(S),13-dihydrolactucopicrin; Lp: lactucopicrin.

SD: standard deviations obtained by three repetitions.

nd: not detected.

**Table 2:** Authentication of CGA and STL in chicory flour by negative-ion UPLC-HRMS analysis.

Metabolites^a^	Formula	M − H+	RDB	RT	M − H+	Precision (ppm)
		theoretical		min	average ± SD	average ± SD
5-CQA	C_16_H_18_O_9_	353.08726	8.5	1.85	353.08856 ± 8.83*E* − 05	5.244 ± 0.250
DHLc	C_15_H_18_O_5_	277.10760	7.5	2.07	277.10884 ± 3.74*E* − 05	6.460 ± 0.135
Lc	C_15_H_16_O_5_	275.09970	8.5	2.30	275.09316 ± 5.10*E* − 05	6.397 ± 0.186
3,5-diCQA	C_25_H_24_O_12_	515.11895	14.5	3.65	515.12061 ± 1.02*E* − 04	4.156 ± 0.260
DHdLc	C_15_H_18_O_4_	261.11260	7.5	4.04	261.11376 ± 4.19*E* − 05	6.209 ± 0.160
dLc	C_15_H_16_O_4_	259.09700	8.5	4.04	259.09803 ± 5.44*E* − 05	6.000 ± 0.197
DHLp	C_23_H_24_O_7_	411.14430	12.5	6.82	411.14657 ± 6.60*E* − 05	6.658 ± 0.160
Lp	C_23_H_22_O_7_	409.12870	13.5	6.91	409.13055 ± 2.46*E* − 04	5.802 ± 0.601

^a^5-CQA: 5-mono-*O*-caffeoylquinic acid; DHLc: 11(S),13-dihydrolactucin; Lc: lactucin; 3,5-diCQA: 3,5-di-*O*-caffeoylquinic acid; DHdLc: 11(S),13-dihydro-8-deoxylactucin; dLc: 8-deoxylactucin; DHLp: 11(S),13-dihydrolactucopicrin; Lp: lactucopicrin.

M − H+: molecular weight without one hydrogen.

RDB: ring double bond.

RT: retention times.

SD: standard deviations obtained by three repetitions.

**Table 3:** Retention time (RT), limit of detection (LOD) and of quantitation (LOQ), linearity range, determination coefficient (*R* ^2^), and regression equation of metabolites tested.

Metabolites^a^	RT	LOD	LOQ	Linearity range	*R* ^2^	Regression equation
	avg ± SD	ng/mL	ng/mL	ng/mL		
5-CQA	1.62 ± 0.010	8.1	8.5	8.5–42,000	0.9993	*y* = 1*E* + 07*x*
DHLc	2.01 ± 0.009	2	5.7	5.7–35,000	0.9930	*y* = 4*E* + 07*x*
Lc	2.29 ± 0.007	6.4	13.7	13.7–70,000	0.9954	*y* = 3*E* + 07*x*
3,5-diCQA	5.11 ± 0.033	9.3	66.5	66.5–62,000	0.9986	*y* = 2*E* + 07*x*
DHdLc	5.41 ± 0.023	9.5	18.1	18.1–50,000	0.9954	*y* = 3*E* + 07*x*
dLc	5.59 ± 0.023	4.1	11.4	11.4–10,000	0.9903	*y* = 6*E* + 07*x*
DHLp	8.74 ± 0.037	2.5	3.3	3.3–2,000	0.9645	*y* = 4*E* + 07*x*
Lp	8.88 ± 0.041	5.9	20.5	20.5–12,000	0.9910	*y* = 3*E* + 07*x*

^a^5-CQA: 5-mono-*O*-caffeoylquinic acid; DHLc: 11(S),13-dihydrolactucin; Lc: lactucin; 3,5-diCQA: 3,5-di-*O*-caffeoylquinic acid; DHdLc: 11(S),13-dihydro-8-deoxylactucin; dLc: 8-deoxylactucin; DHLp: 11(S),13-dihydrolactucopicrin; Lp: lactucopicrin.

**Table 4:** Extractability rate of the extraction H_2_O/CHCl_3_/MeOH 30/30/40 (v/v/v), repeatability, and reproducibility expressed in terms of the variation (RSD %) of contents. The repeatability and reproducibility of the HPLC-DAD method are expressed in terms of the variation (RSD %) of retention times (RT) and areas.

	Metabolites^a^							
	5-CQA	3,5-diCQA	DHLc	Lc	DHdLc	dLc	DHLp	Lp
**Extraction**								
Extractability rate %								
Root	94	76	79	82	85	86	84	85
Flour	82	82	85	84	85	84	85	85
Roasted grains	86	85	88	89	89	87	89	89
Repeatability RSD %								
Root	±4	±6	±2	±2	±7	±9	±6	±8
Flour	±6	±5	±9	±7	±6	±7	±5	±7
Roasted grains	±4	±4	±2	±10	±7	±5	±10	±4
Reproducibility RSD %								
Root	±6	±6	±5	±4	±9	±11	±8	±8
Flour	±5	±6	±7	±10	±7	±9	±7	±11
Roasted grains	±6	±6	±9	±8	±11	±5	±12	±6

**HPLC-DAD analysis**								
Repeatability								
RT RSD %								
Root	±0	±0.6	±0	±0	±0.3	±0.9	±0.6	±0.6
Flour	±0.4	±0.6	±0.4	±0.4	±0.5	±0.9	±0.4	±0.3
Roasted grains	±0.3	±0.6	±0.3	±0.3	±0.6	±0.8	±0.6	±0.7
Areas RSD %								
Root	±5.1	±7.2	±5.7	±3.5	±9.7	±14.3	±11.3	±4.1
Flour	±1.3	±1.6	±3.2	±2.9	±6.6	±12	±13.2	±6.6
Roasted grains	±2.5	±4.1	±7.7	±1.1	±6.1	±3.3	±1.3	±1.1
Reproducibility								
RT RSD %								
Root	±0.6	±1.5	±1.0	±1.0	±1.0	±0.6	±1.5	±1.0
Flour	±0.6	±0.6	±0	±0.6	±0.6	±1	±1.2	±0.6
Roasted grains	±0.6	±1.0	±0	±1.0	±1.2	±0.6	±1.2	±1.5
Areas RSD %								
Root	±11.5	±7.7	±2.7	±11.9	±8.3	±6.4	±10.5	±10.2
Flour	±5.4	±3.6	±5.5	±3.6	±3.8	±9.5	±6.2	±4.9
Roasted grains	±4.4	±5.8	±8.8	±3.3	±6.7	±8.4	±5.1	±1.8

^a^5-CQA: 5-mono-*O*-caffeoylquinic acid; 3,5-diCQA: 3,5-di-*O*-caffeoylquinic acid; DHLc: 11(S),13-dihydrolactucin; Lc: lactucin; DHdLc: 11(S),13-dihydro-8-deoxylactucin; dLc: 8-deoxylactucin; DHLp: 11(S),13-dihydrolactucopicrin; Lp: lactucopicrin.

RSD: respective standard deviations.

**Table 5:** Recovery (%) of the extraction of H_2_O/CHCl_3_/MeOH 30/30/40 (v/v/v) with different additions (5-CQA, 3,5-diCQA, and DHLc) to flour.

	Metabolites^a^		
	5-CQA	3,5-diCQA	DHLc
Add 5-CQA			
0.075 mM	96%	—	—
0.15 mM	97%	—	—
0.3 mM	94%	—	—
Add 3,5-diCQA			
0.0075 mM	—	106%	—
0.03 mM	—	111%	—
0.075 mM	—	116%	—
Add DHLc			
0.007 mM	—	—	106%
0.014 mM	—	—	103%
0.045 mM	—	—	122%

^a^5-CQA: 5-mono-*O*-caffeoylquinic acid; 3,5-diCQA: 3,5-di-*O*-caffeoylquinic acid; DHLc: 11(S),13-dihydrolactucin.
